# Circulation of RSV Subtypes A and B Among Mexican Children During the 2021–2022 and 2022–2023 Seasons

**DOI:** 10.3390/pathogens14100996

**Published:** 2025-10-02

**Authors:** Selene Zárate, Blanca Taboada, Karina Torres-Rivera, Patricia Bautista-Carbajal, Miguel Leonardo Garcia-León, Verónica Tabla-Orozco, María Susana Juárez-Tobías, Daniel E. Noyola, Pedro Antonio Martínez-Arce, Maria del Carmen Espinosa-Sotero, Gerardo Martínez-Aguilar, Fabian Rojas-Larios, Alejandro Sanchez-Flores, Carlos F. Arias, Rosa María Wong-Chew

**Affiliations:** 1 Posgrado en Ciencias Genómicas, Universidad Autónoma de la Ciudad de México, Ciudad de México 03100, Mexico; karinatorresrivera8@gmail.com; 2 Departamento de Genética del Desarrollo y Fisiología Molecular, Instituto de Biotecnología, Universidad Nacional Autónoma de México, Cuernavaca 62210, Mexico; blanca.taboada@ibt.unam.mx (B.T.); carlos.arias@ibt.unam.mx (C.F.A.); 3 Infectious Diseases Research Laboratory, Research Division, Facultad de Medicina, Universidad Nacional Autónoma de México, Ciudad de México 04510, Mexico; pbautistac@comunidad.unam.mx (P.B.-C.); mlgl7@unam.mx (M.L.G.-L.); 4 Hospital Pediátrico de Coyoacán, Ciudad de México 04100, Mexico; vtablaorozco@gmail.com; 5 Hospital Central “Dr. Ignacio Morones Prieto”, San Luis Potosí 78210, Mexico; susana.juarez@uaslp.mx; 6 Centro de Investigación en Ciencias de la Salud y Biomedicina, Universidad Autónoma de San Luis Potosí, San Luis Potosí 78210, Mexico; dnoyola@uaslp.mx; 7 Antiguo Hospital Civil de Guadalajara Fray Antonio Alcalde, Guadalajara 44280, Mexico; pama130@yahoo.com.mx; 8 Hospital General de México “Dr. Eduardo Liceaga”, Ciudad de México 06720, Mexico; carmenespinosa6@hotmail.com; 9 Hospital Municipal del Niño de Durango, Durango 34167, Mexico; uimec@yahoo.es; 10 Facultad de Medicina y Nutrición, Universidad Juárez del Estado de Durango, Durango 34000, Mexico; 11 Hospital Regional IMSS Bienestar Colima, Colima 28085, Mexico; frojas@ucol.mx; 12 Unidad Universitaria de Secuenciación Masiva y Bioinformática, Instituto de Biotecnología, Universidad Nacional Autónoma de México, Cuernavaca 62210, Mexico; alejandro.sanchez@ibt.unam.mx

**Keywords:** RSV circulation, viral phylogenetics, virus epidemiology

## Abstract

Respiratory syncytial virus (RSV) remains a leading cause of pneumonia in young children in Mexico and worldwide. To investigate RSV dynamics in Mexico, we conducted a multicenter study from August 2021 to July 2023 in six hospitals across five States, analyzing respiratory samples from children under five years with pneumonia. Multiplex RT-PCR identified 203 RSV-positive cases, of which 123 were RSV-B and 80 RSV-A. Interestingly, 77% of the collected samples showed evidence of coinfection with other respiratory pathogens, with rhinovirus, *Haemophilus influenzae*, and *Streptococcus pneumoniae* being the most common. Also, RSV-B dominated in 2021–2022, whereas RSV-A prevailed in 2022–2023, mirroring trends observed in the United States. Sequences of the genes encoding G and F proteins showed that RSV-A lineages were more diverse, with A.D.1, A.D.1.8, and A.D.5.2 being frequently detected. In contrast, nearly all RSV-B sequences belonged to lineage B.D.E.1. Finally, ancestral state inference suggests repeated introductions from the USA and other North American countries, with limited evidence of sustained local circulation. These findings show different trends in RSV circulation between two consecutive seasons and the importance of genomic surveillance to monitor RSV diversity, evaluate vaccine impact, and inform public health strategies in Mexico’s evolving post-pandemic respiratory virus landscape.

## 1. Introduction

Respiratory syncytial virus (RSV) is a major contributor to severe acute respiratory disease in young children and the elderly. Two serologically distinct subtypes, A and B, of RSV have been established. While some studies have associated RSV-A with a more severe clinical presentation than RSV-B, many have not found a significant difference [1]; instead, both typically co-circulate each year. A global seasonal circulation pattern has been reported, in which RSV infections originate in the tropics at the end of summer and migrate toward higher latitudes during winter [2]. However, these circulation patterns were disrupted by the COVID-19 pandemic. In a survey of 11 countries, the expected RSV epidemic of winter 2020–2021 suffered a 39-week delay in the onset of the RSV season, resulting in a summer outbreak in areas where winter epidemics are typically observed [3]. Moreover, surveillance data from 2021 and 2022 in the United States revealed significant changes. An atypical summer outbreak in 2021 was mainly associated with RSV-B, while in 2022, an earlier-than-usual epidemic was dominated by RSV-A and was accompanied by a higher number of pediatric hospitalizations compared with previous years [4]. The reasons behind these shifts are not fully understood, although non-pharmacological interventions implemented to control SARS-CoV-2 may have contributed to modifying the circulation of other respiratory pathogens.

In Mexico, RSV has consistently been identified as one of the most prevalent respiratory pathogens in children. Hospital-based studies from the 2022–2023 and 2023–2024 winter seasons documented RSV as a major cause of severe lower respiratory tract infections and hospitalizations, representing more than 40% of pediatric admissions during peak circulation periods [5,6].

These recent findings align with earlier evidence showing that RSV has long been a dominant cause of pediatric respiratory infections in Mexico. Data collected between 2010 and 2014 reported that RSV-A accounted for more than 70% of cases nationally, although no genomic subtyping was performed [7]. Additional reports emphasized RSV’s contribution to intensive care unit admissions and mortality in infants [8] as well as its impact on the hospitalization of preterm infants [9]. Other studies have described the epidemiology and clinical features of RSV in single hospitals [10]. At the same time, regional data reported alternating circulation with influenza [11] and the contribution of RSV, influenza, and parainfluenza viruses to acute respiratory infections [12].

Recent national consensus guidelines highlight the importance of RSV surveillance in Mexico [13]. However, these initiatives rely primarily on clinical and epidemiological data. At the same time, genomic information remains scarce, with only recent work describing the dominance of A.D lineages in RSV-A and B.D.E.1 in RSV-B between 2021 and 2024 [14,15]. Comprehensive genomic analyses covering multiple hospitals and regions are still lacking, making it challenging to assess subtype turnover, viral introductions, and the relationship with changes in clinical burden.

In this study, we present a multicenter analysis of RSV circulation in Mexico during two consecutive seasons (2021–2022 and 2022–2023) by combining epidemiological, clinical, and genomic data from six hospitals across five states, and analyzing the G and F genetic sequences of RSV-positive samples, as it has been reported that when these markers are used together, they have a misclassification rate of 0.07% for RSV-A and none for RSV-B [16]. Overall, we provide novel insights into subtype dynamics, lineage diversity, and viral introductions.

## 2. Materials and Methods

### 2.1. Study Design and Population

A cross-sectional longitudinal epidemiological study was conducted in six hospitals during the pandemic to investigate the viruses and bacteria associated with pneumonia in children, from July 2021 to June 2023. These included Hospital Pediátrico de Coyoacán, Antiguo Hospital Civil de Guadalajara “Fray Antonio Alcalde”, Guadalajara, Jalisco; Hospital Central “Dr. Ignacio Morones Prieto”, San Luis Potosí; Hospital General de México “Dr. Eduardo Liceaga”, Hospital Municipal del Niño de Durango, Hospital Regional IMSS Bienestar Colima

### 2.2. Case Definition, Inclusion, and Exclusion Criteria

Participant selection included children younger than 5 years old with a clinical and/or radiological diagnosis of pneumonia. A “pneumonia case” was defined as the presence of respiratory symptoms, including respiratory distress, cough, tachypnea, cyanosis, with or without fever within the first week of symptom onset, and/or a chest X-ray showing the presence of pulmonary infiltrates. Exclusion criteria included inadequate respiratory sample or lack of clinical and demographic data. All demographic and clinical characteristics were collected using a specially designed format for the study.

### 2.3. Sample Collection

Patients who met the inclusion criteria and attended the emergency department or were hospitalized at the participating hospitals had their parents invited to have their children participate in the study. Once informed consent was obtained, and data collection was completed, the sample was obtained through a nasal swab. The samples were placed in a viral transport medium, frozen at −70 °C, and sent to the Infectious Diseases Research Laboratory (LIEI), School of Medicine, UNAM, where they were stored at −70 °C until processing.

### 2.4. Multiplex RT-PCR for the Detection of Respiratory Viruses

The respiratory samples were processed at the Infectious Diseases Research Laboratory, Faculty of Medicine, UNAM, using the Allplex™ SARS-CoV-2/FluA/FluB/RSV Assay and Allplex™ Respiratory Full Panel multiplex RT-PCR kits (Seegene, Seoul, Republic of Korea) according to the manufacturer’s instructions. Total RNA was extracted with the STARMag™ 96 × 4 Universal Cartridge kit on the Seeprep 32 system. For the PCR reaction, 17 µL of One-Step RT-PCR Mastermix was dispensed into each tube of an 8-strip white tube, and 8 µL of nucleic acid extract was added to each reaction. Multiplex PCR was performed using a CFX96 real-time thermocycler (Bio-Rad, Hercules, CA, USA) as described by Seegene, followed by automatic analysis of the results with the Seegene Viewer V2.1 software (Seegene, Seoul, Republic of Korea). The combination of these assays simultaneously detects 29 respiratory viruses—including SARS-CoV-2, Influenza A subtypes (H1, H1N1pdm09, H3) and B; parainfluenza virus types 1–4; adenovirus; bocavirus; respiratory syncytial virus A and B; human metapneumovirus (A/B); coronaviruses NL63, 229E, and OC43; rhinovirus; and enterovirus—as well as seven bacterial respiratory pathogens (*Mycoplasma pneumoniae*, *Bordetella pertussis*, *B. parapertussis*, *Chlamydophila pneumoniae*, *Haemophilus influenzae*, *Streptococcus pneumoniae*, and *Legionella pneumophila*). The Seegene Viewer V3.33.002 software automatically interprets amplification curves, considering as positive those respiratory targets that generate adequate exponential fluorescence with Ct values < 42 cycles, and reports results for all included pathogens together with the internal control (IC).

In this study, 203 patients with a positive RSV test result were included. Metadata from the samples, including all pathogens that tested positive in each sample, the patient’s age, and sex, were collected. Sample metadata is provided in Supplementary Table S1. Using the Upset package v.1.4.0 [17] in R v.4.3.0 [18], an upset plot was created for the five most frequently detected pathogens to determine if a typical coinfection pattern existed. Additionally, bar plots to show the distribution of samples over time were built using ggplot2 v.3.5.2 [19] in R. Epidemiological data for RSV infections in Mexico were obtained from FluNet (https://www.who.int/tools/flunet), retrieved on 3 June 2025.

### 2.5. Genotyping and Sequencing

For 105 samples, Illumina sequencing was performed using a whole-genome amplicon approach on the NextSeq 500 platform (Illumina, San Diego, CA, USA) with a 300-cycle kit, in a paired-end configuration, to produce reads of 150 bp in length. Raw reads were processed with fastp v0.23.4 [20], which applied quality trimming and removed duplicate reads. Filtered reads were mapped to the RSV-A or RSV-B reference genomes using Bowtie2 v2.4.5 [21], and the alignments were handled with SAMtools v1.16 [22]. Consensus genomes were generated with iVar v1.4 [23] at a minimum depth threshold of 10×. The genomes were generated as part of the viral surveillance efforts carried out by the Mexican Consortium for Genomic Surveillance (CoViGen-Mex). The sequences were submitted to the GISAID Epi-RSV database and can be retrieved with ID EPI_SET_250925pf; individual accession numbers are listed in Supplementary Table S2.

From the obtained genomes, only the sequence of the region covering genes G and F of RSV was used for further analyses. Of these sequences, 34 corresponded to subtype A and 71 to subtype B. Each subtype was aligned using MAFFT v7 [24] with all high-quality sequences available from the GISAID-EpiRSV database [25,26], with a collection date prior to June 2023. The resulting alignments were reduced using cd-hit v.4.8.1 [27] to eliminate identical sequences, resulting in alignments containing 1831 sequences for RSV-A and 2138 for RSV-B. The downloaded sequences can be accessed with the number EPI_SET_250828fk. The alignments were analyzed using Nextclade [28] to determine the lineages of the sequences.

A maximum likelihood tree for each RSV subtype was reconstructed using iqtree2 v.2.2.2.6 [29], with the substitution model, GTR+F+I+R4 for RSV-A and GTR+F+I+R5 for RSV-B, as determined by ModelFinder v.1.0 [30]. The resulting phylogeny was time-scaled according to the collection date with LSD2 v.2.3 [31]. The phylogeny was visualized in R v.4.3.0 using the package ggtree v.3.8.2 [32]. To determine the geographical origin of the Mexican sequences, an ancestral reconstruction of the location was carried out using PASTML v.1.9.15 [33].

## 3. Results

### 3.1. Sample Data Analysis

**Figure 1:**
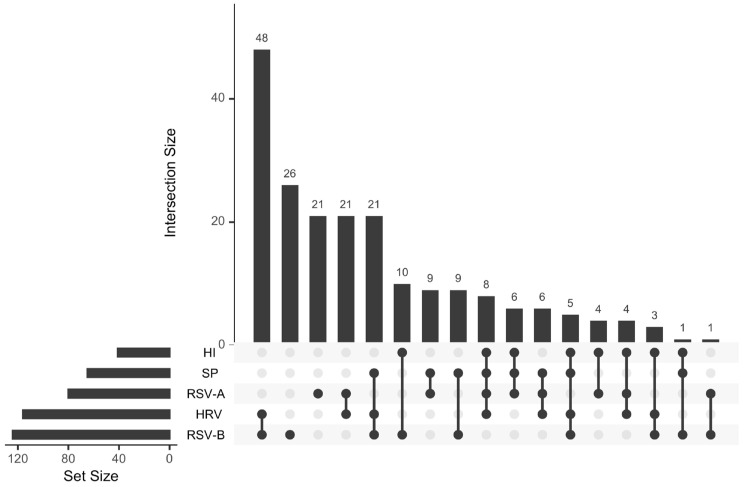
An upset plot shows the pattern of coinfections among the five most frequently detected pathogens in the test. The lower left bar plot shows the number of children with a positive test for each of the pathogens. The main bar plot displays the number of children who tested positive for the pathogens indicated in the diagram below. HI: *Haemophilus influenzae*, SP: *Streptococcus pneumoniae*, RSV-A: Respiratory syncytial virus A, HRV: Human rhinovirus, RSV-B: Respiratory syncytial virus B.

In this study, we analyzed samples from 203 children with positive RSV test, of which 123 were positive for RSV-B and 80 for RSV-A. A large proportion of children showed evidence of coinfection with other pathogens. Overall, 79.8% of RSV-positive cases harbored at least one additional agent, with similar frequencies between subtypes (78.7% in RSV-A and 80.5% in RSV-B). Most coinfections involved RSV in combination with one other pathogen (50.3%), while 21.7% included two, and 7.9% three or more additional pathogens. As shown in Figure 1, only 26 children were solely infected with RSV-B and 21 with RSV-A, whereas several presented with up to four pathogens. Rhinovirus was the most common co-detected virus, while *Haemophilus influenzae* and *Streptococcus pneumoniae* were the most frequent bacterial agents. Coinfection frequencies varied between 71% and 100% across epidemic months and between 67% and 100% across states, as shown in Supplementary Tables S1.

**Figure 2:**
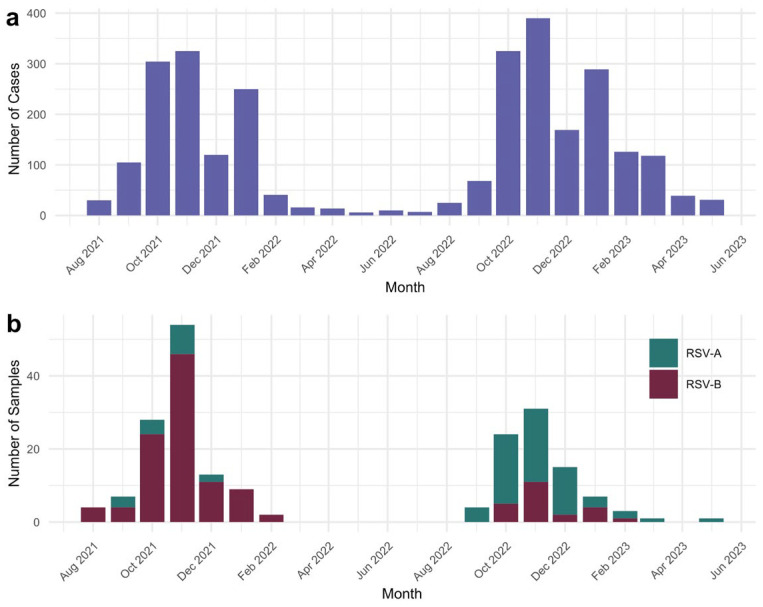
Distribution of RSV-positive cases and samples during the study period. (**a**) Number of nationwide RSV reported cases in Mexico according to data from FluNet, corresponding to the sampling period. (**b**) Number of RSV-positive samples per month, divided by subtype.

The samples were obtained during two seasons of RSV circulation, the first one from August 2021 to February 2022, and the second from September 2022 to May 2023. For context, the number of nationwide reported cases is shown in Figure 2a, according to data retrieved from FluNet, which only reports the number of cases positive for RSV, but does not include subtyping information. Figure 2b shows the RSV-positive samples from this study by collection date, including subtype. As shown in Figure 2, the sampling periods correspond to the epidemic peaks. In the first season, RSV-B dominates, whereas a higher prevalence of RSV-A can be observed in the second season. Although there is no national data to compare the relative prevalence of RSV subtypes, this observation agrees with data from the USA [4].

**Figure 3:**
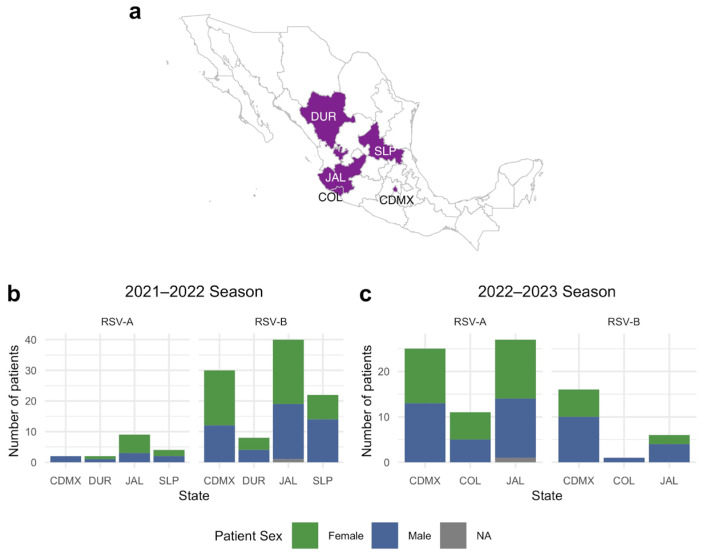
Geographic distribution and number of patients from each state by sex, divided by RSV subtype. (**a**) Map showing the States where the samples were taken. (**b**) Patients with a positive RSV A or B test from August 2021 to February 2022. (**c**) Patients with a positive RSV A or B test from September 2022 to May 2023.

The samples were retrieved from five States of Mexico: Mexico City, Jalisco, Colima, Durango, and San Luis Potosí. A map showing the location is presented in Figure 3a. Figure 3b,c show the distribution of RSV in each State during the 2021–2022 and 2022–2023 seasons, respectively. As can be observed in Figure 3b, approximately the same number of samples were obtained from female and male patients (51% female) during the 2021–2022 season. In contrast, during the 2022–2023 season (Figure 3c), a greater proportion of samples were from male patients (45% female), but not too different from the previous year. Additionally, in both seasons, most samples were from Mexico City (27 and 48%) and Jalisco (42 and 38%).

### 3.2. RSV Lineage Determination

All the samples were subjected to sequencing; however, given that some of the samples had a low viral load (CT > 28), only 34 sequences for RSV-A and 71 sequences for RSV-B were obtained. The recovered sequences were classified using Nextclade to determine the virus lineages present in the samples. Among the RSV-A sequences, five different lineages were identified. Both A.D.1 and A.D.1.8 were detected in the two seasons, and their frequencies increased notably from 3.1% and 4.7% in 2021–2022 to 17.1% and 24.4% in 2022–2023, respectively. Interestingly, lineage A.D.5.2 appeared exclusively during the 2022–2023 season, representing 17.1% of the sequences, whereas A.D.3 and A.D.3.3 were detected sporadically. In contrast, RSV-B sequences showed little diversity, with 98.6% corresponding to lineage B.D.E.1 and only one sample classified as B.D.4.1.1. These results, summarized in Table 1, highlight the higher lineage diversity and temporal shifts observed in RSV-A compared with the marked predominance of a single lineage in RSV-B and are consistent with previous reports [14,15]. Additionally, the lineage distribution by state is also shown in Table 1. Although there are too few samples to draw definitive conclusions about differences in lineage distribution, some trends can be observed. There are too few RSV-A sequences from season 2021–2022 to observe any patterns; however, in season 2022–2023, we can observe that A.D.1 was sampled mostly in Colima (six samples), while the most frequently sampled lineage in Jalisco was A.D.1.8. On the other hand, two lineages were detected at similar rates in Mexico City.

### 3.3. Phylogenetic Analysis

**Figure 4:**
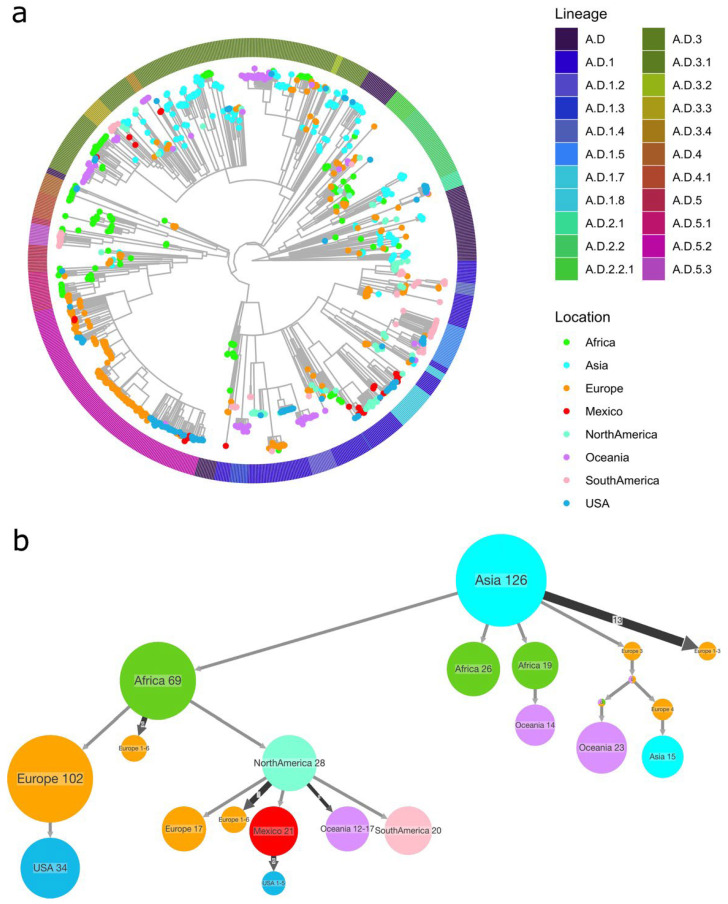
RSV-A phylogenetic analysis and ancestral state analysis. (**a**) A maximum likelihood time-scaled tree constructed using the region coding for G-F proteins, with the GTR+F+I+I+R4 substitution model. The tips are colored by sampling region, and the colors in the outside ring indicate the assigned lineage. (**b**) Compressed visualization of the ancestral state reconstruction for the sampling site of RSV-A, colored by geographical region.

A phylogenetic reconstruction was carried out to determine the relationship between Mexican sequences and those reported in GISAID from all over the world. Figure 4a shows the phylogeny of RSV-A. For simplicity, the tips of the tree are colored according to the continent of sampling, except sequences from Mexico (shown in red) and the USA (in blue), as we hypothesized a relation between Mexican sequences and those from the USA. As can be observed in the tree (Figure 4a), Mexican sequences mostly cluster with those from the USA or other countries in North America. However, clustering with European sequences can be observed in one group. This distribution suggests that Mexican sequences are not monophyletic but rather scattered, supporting the hypothesis of multiple introductions rather than sustained local circulation. Interestingly, sequences from lineages A.D.1 and A.D.1.8, which circulate in both seasons, do not cluster together, suggesting new introductions into Mexico and no local continuous circulation.

To further explore the history of RSV-A’s introductions into Mexico, an ancestral state reconstruction of the sample’s location was carried out using PastML v.1.9.15. Figure 4b shows the compressed version of the reconstruction, where the point of origin of the Mexican sequences is in North America. Some ancestral nodes also connect with Europe and Asia, indicating occasional introductions from other regions. Together, these results highlight that RSV-A diversity in Mexico is shaped by repeated international introductions, mainly from the USA and neighboring countries, rather than by long-term local persistence. A full version of the tree is available in Supplementary Figure S1.

**Figure 5:**
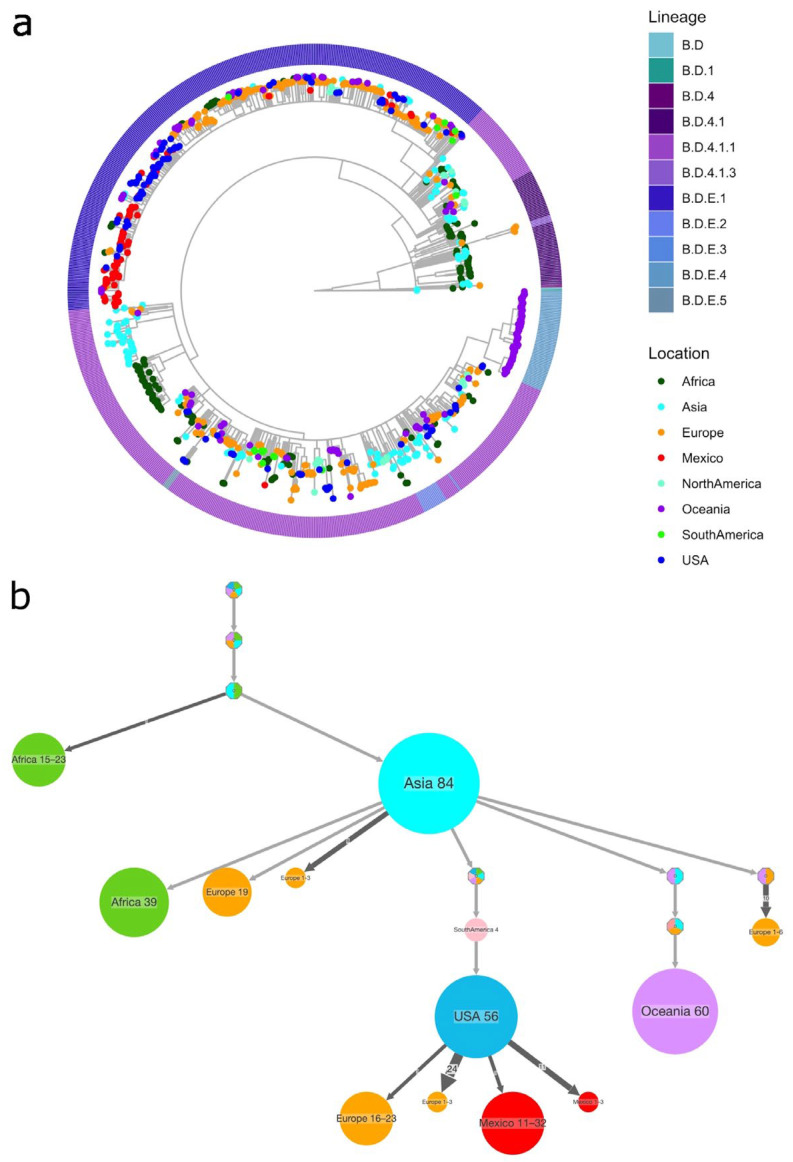
RSV-B phylogenetic reconstruction. (**a**) A maximum likelihood time-scaled tree was built using the region coding for G-F proteins, with the GTR+F+I+I+R5 substitution model. The tips are colored by sampling region, and the colors in the outside ring indicate the assigned lineage. (**b**) Compressed visualization of the ancestral state reconstruction of the sampling site for RSV-B, colored by geographical region.

Similarly, a phylogenetic reconstruction was performed for RSV-B, as shown in Figure 5a, where the tips are colored by continent. Sequences from Mexico are highlighted in red, and those from the USA are in blue to distinguish them from the rest. Mexican sequences were predominantly assigned to lineage B.D.E.1 and clustered closely with US sequences, reflecting the limited genetic diversity of this subtype compared with RSV-A. Several large clusters composed mainly of Mexican sequences indicate local chains of transmission, although the overall topology suggests that multiple introductions occurred.

The PastML reconstruction further supports this pattern (Figure 5b and Figure S2), with most Mexican sequences tracing back to the USA and North America, but also receiving additional contributions from other regions, such as Asia and Europe. These findings suggest that RSV-B diversity in Mexico during the study period resulted from repeated introductions, primarily from the USA, followed by local spread within the country. The full version of the tree is provided in Supplementary Figure S2.

## 4. Discussion

In this study, we report a remarkably high frequency of a pattern of commonly occurring coinfections in RSV-positive pediatric samples. Using an upset plot to analyze coinfection patterns with other respiratory pathogens could shed light on how RSV interacts with different viruses. A limitation of these results is that the pathogens were detected only using nucleic acid-based tests, so a distinction between colonization and infection cannot be fully ascertained. Nevertheless, other studies have reported a higher tendency to detect coinfections of respiratory pathogens in children than in adults [34], but with a coinfection rate of 35%, lower than the rate reported here. The most frequent codetections found involved rhinovirus, *Haemophilus influenzae*, and *Streptococcus pneumoniae*, consistent with previous observations of viral–viral and viral–bacterial interactions. However, the complexity of these interactions remains largely unexplored. Still, it has been reported that rhinoviruses and RSV are negatively correlated [35]. Interestingly, other studies have found negative correlations between rhinovirus and influenza, as well as positive correlations between RSV and metapneumovirus [36]. Finally, the relation between RSV and bacteria has been studied frequently, but the mechanisms behind this phenomenon remain unknown [37]. The contrasting evidence underscores the complexity of pathogen interactions in the respiratory tract. Further studies using approaches such as upset plots or network analyses may help clarify these relationships, which are clinically relevant for diagnosis, treatment strategies, and the management of respiratory infections in children.

Beyond the pathogen codetection analysis, our data highlight the differences in the circulation patterns of RSV from one season to the next. Notably, during the 2021–2022 season, RSV-B dominated, and a shift was observed in the following season. This finding is consistent with trends observed in other regions [3,4], suggesting that the COVID-19 pandemic may have altered the dynamics of RSV transmission, as evidenced by the shift in the peak towards summer in 2021. Due to the limited epidemiological information available in Mexico from previous years, it is challenging to assess the changes in RSV dynamics. Still, as the analysis of the two seasons reported in this study suggests, Mexico’s subtypes circulation and seasonality resemble those reported from the USA; therefore, it is reasonable to assume that this has been historically the case. Interestingly, a study from Italy reported dominance of RSV-A in 2021–2022 and of RSV-B in 2022–2023 [38]. This opposite trend shows that local circulation patterns are possible, and generalizations should be performed with care, as many reports do not distinguish between RSV subtypes, making it difficult to obtain a clearer picture of the trends in subtype dominance.

The changes in RSV and other respiratory pathogen circulation are due to non-pharmacological interventions implemented during the COVID-19 pandemic, such as mask-wearing and social distancing. Using data from European countries, a model showed an increase in the proportion of RSV-A in the post-pandemic years [25], with data suggesting an advantage for this virus subtype that may persist long after restrictions have been lifted [27]. The drastic shifts in subtype prevalence and seasonality post-pandemic raise questions about the long-term effects of such interventions on the epidemiology of respiratory viruses.

The genome sequencing results reported here revealed the presence of distinct genetic lineages of RSV-A and RSV-B in Mexico. Notably, RSV-A exhibits more diversity, with A.D.1 and A.D.1.8 detected during both seasons, and A.D.5.2 present only in the 2022–2023 season. However, there is limited information to shed light on regional differences in lineage distribution for RSV-A. Still, it is remarkable that during the 2022–2023 season, the three sampled states show a vast difference in the lineages detected, suggesting an overall high diversity throughout Mexico. The higher diversity of RSV-A reflects global data [39], suggesting that multiple introductions resulted in the circulation of different lineages, possibly accompanied by the maintenance of variants from previous seasons.

In contrast, RSV-B showed less diversity, as nearly all the sequences corresponded to genotype B.D.E.1. This trend is also global, with B.D.E.1 beginning its growth in 2021 and rapidly replacing other lineages [40]. This clade replacement may be due to a growth advantage of the new variant or a consequence of the limited RSV circulation resulting from mask-wearing and social distancing policies during the COVID-19 pandemic, which created a bottleneck effect. Still, since sequence data from previous years in Mexico are minimal, the origin of these differences is difficult to assess, and it is challenging to determine the dynamics of lineage distribution in the country.

Despite the insights into RSV circulation in Mexico presented in this study, it is essential to address its limitations. Firstly, the number of samples is small, and only five states out of 32 were included. Additionally, most of the samples are from hospitalized children, which may introduce a bias in the sequenced viruses. Therefore, our sample may not reflect the diversity of RSV circulating in Mexico, particularly if regional differences occur.

As sequencing data accumulate, analysis may reveal regional patterns of RSV distribution and provide insights into the introduction of novel variants. So far, we have identified a close relationship with sequences from the USA and North America, with most of these sequences originating from Canada. These viruses are likely being repeatedly introduced into Mexico. Understanding the genetic diversity of RSV and its dynamics will be crucial to measure the impact of the newly approved vaccines, future vaccine development, and public health strategies.

## Figures and Tables

**Table 1:** RSV lineages found in the sequenced samples by state and season of sampling.

Lineage	Season 2021–2022					Season 2022–2023			
	CDMX	DUR	JAL	SLP	Total (%)	CDMX	COL	JAL	Total (%)
**RSV-A**									
A.D.1	2	0	0	0	2 (3.1)	0	6	1	7 (17.1)
A.D.1.8	0	1	2	0	3 (4.7)	4	0	6	10 (24.4)
A.D.3	0	1	1	0	2 (3.1)	0	0	1	1 (2.4)
A.D.3.3	0	0	2	0	2 (3.1)	0	0	0	0
A.D.5.2	0	0	0	0	0	5	1	1	7 (17.1)
**RSV-B**									
B.D.E.1	17	5	19	13	54 (84.4)	12	1	3	16 (39.0)
B.D.4.1.1	0	0	1	0	1 (1.6)	0	0	0	0

## Data Availability

All sequences generated were shared through GISAID-EpiRSV with ID EPI_SET_250925pf. Also, other sequences used in this study were retrieved from GISAID-EpiRSV and can be accessed with EPI_SET_250828fk.

## Supplementary Materials

The following supporting information can be downloaded at: https://www.mdpi.com/article/10.3390/pathogens14100996/s1, Figure S1: RSV-A PASTML reconstruction; Figure S2: RSV-B PASTML reconstruction; Table S1: Patient metadata. Table S2: Reported sequences accession numbers.
