# Supplementary figures and images for: Circulation of RSV Subtypes A and B Among Mexican Children During the 2021–2022 and 2022–2023 Seasons
Source: Pathogens. 2025 Oct 2;14(10):996. doi: 10.3390/pathogens14100996 (PMC12567045; doi:10.3390/pathogens14100996)

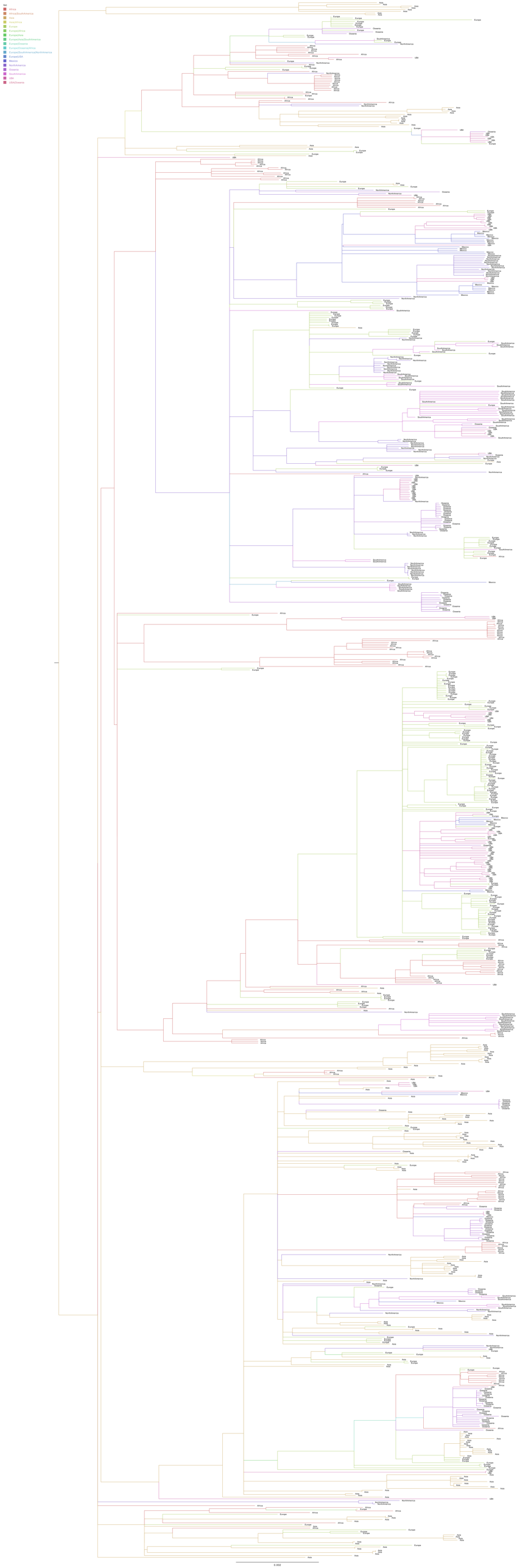

Supplement: Supplementary file 1 [file pathogens-14-00996-s001.zip › Figure S1_RSVAFull_PASTML.pdf]

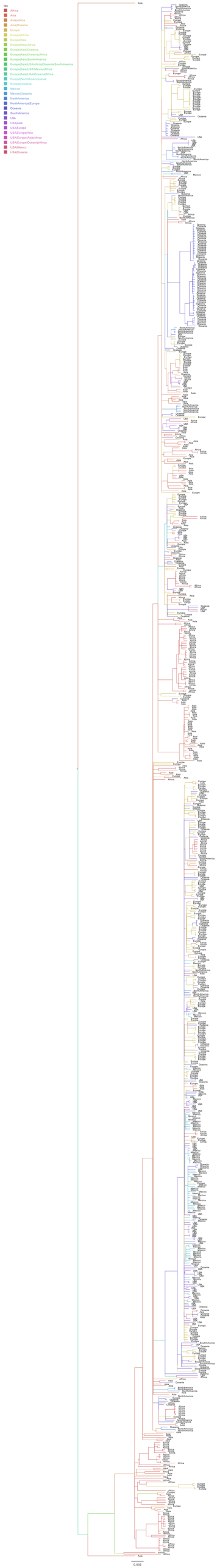

Supplement: Supplementary file 1 [file pathogens-14-00996-s001.zip › Figure S2_SVBGF_PASTMLfull.pdf]
